# The Life and Science of Professor Tsuneko Okazaki, and her time at Fujita Health University

**DOI:** 10.20407/fmj.2023-014

**Published:** 2023-11-29

**Authors:** Johannes M. Dijkstra, Toshiharu Nagatsu

**Affiliations:** 1 Office of Research Administration, Fujita Health University, Toyoake, Aichi, Japan; 2 Center for Research Promotion and Support, Fujita Health University, Toyoake, Aichi, Japan; 3 Adviser, Fujita Health University, School of Medicine, Toyoake, Aichi, Japan

**Keywords:** Professor Tsuneko Okazaki, Professor Reiji Okazaki, DNA replication, Okazaki fragments, Fujita Health University

## Abstract

Distinguished Professor Emeritus Tsuneko Okazaki is a hero of science. Together with her late husband, Professor Reiji Okazaki, she discovered that DNA replication involves the discontinuous synthesis of the DNA lagging strand by intermediates of, what is now called, “Okazaki fragments.” She has been a pioneer for women in science and, in 1983, became the first female full Professor at Nagoya University. From 1997 to 2012, she was a full Professor and later a Visiting Professor at Fujita Health University, and this review zooms in on that period. Besides a summary of her career, this article also includes personal memories of researchers who worked with Professor Okazaki.

## Okazaki, a name known throughout the world

**Figure 1:**
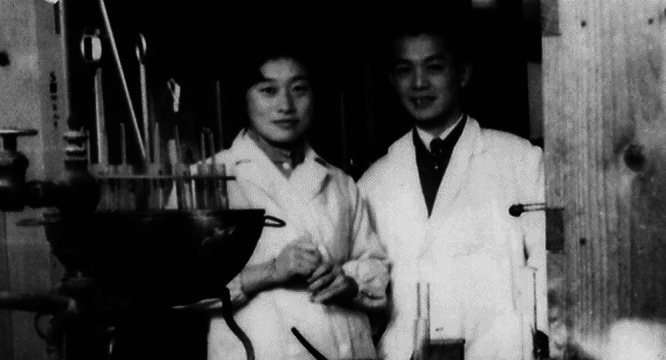
Tsuneko and Reiji in the lab, 1958. The photograph was provided courtesy of Nagoya University.

**Figure 2:**
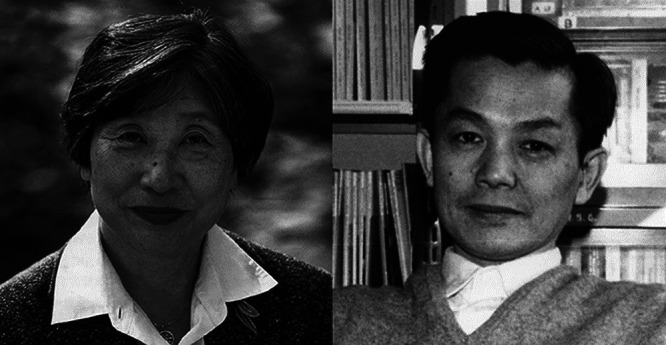
Portrait photographs of Tsuneko and Reiji (taken at different times). The photographs were provided courtesy of Nagoya University.

**Figure 3:**
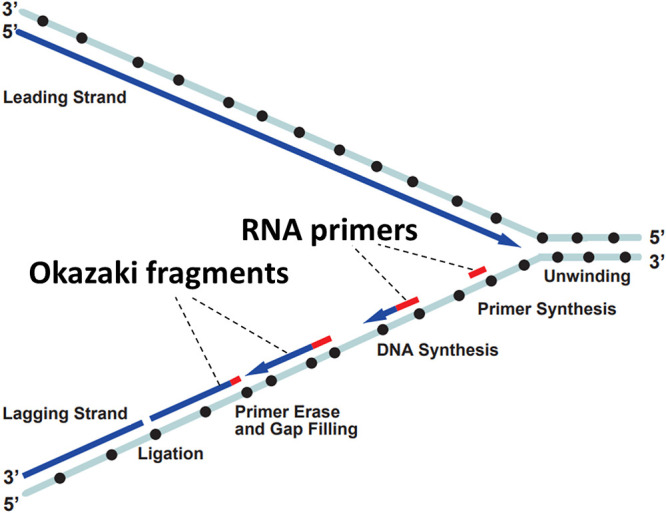
Steps of the discontinuous DNA replication reaction. The leading strand is synthesized continuously while the lagging strand is synthesized discontinuously. The elongation reaction of the lagging strand consists of five steps: I, Unwinding of the DNA template; II, Primer synthesis; III, DNA (Okazaki fragment) synthesis; IV, Primer degradation and gap filling; and V, Ligation of Okazaki fragments. The dots on the template DNA indicate the signal sequences for primer RNA synthesis. The figure and legend are, with a slight modification in the figure, from Figure 11 in reference.^1^

Many people worldwide, during high school biology classes or at university, have heard the last name of Distinguished Professor Emeritus Tsuneko Okazaki. Together with her late husband, Professor Reiji Okazaki (1930–1975) (Figures 1 and 2), she discovered that the “lagging strand” of the replicating DNA helix is not immediately synthesized as a continuous chain but first as small fragments (Figure 3).^1^ They presented these findings in 1968 at a Cold Spring Harbor Symposium, where, for these fragments, Rollin D. Hotchkiss coined the term “Okazaki pieces.”^2^ Later, these became more commonly known as “Okazaki fragments.”

Reiji and Tsuneko are heroes of science and, Tsuneko, additionally, is known for her pioneering role in representing women in science.

After Professor Tsuneko Okazaki retired from Nagoya University, she became a full Professor (1997–2002) and Visiting Professor (2002–2012) at the Institute for Comprehensive Medical Science of Fujita Health University (FHU) (Table 1). In August 2022, FHU held a webinar event in her honor, with Professor Fuyuhiko Tamanoi of Kyoto University and the University of California, Los Angeles—a former student of hers who has been very successful in his own right—as a guest speaker. The present article is also dedicated to her and provides an overview of her life and work while zooming in on her period at FHU.

## Early life and marriage

Tsuneko was born as Tsuneko Hara in Moriyama Ward, Nagoya, 1933, with an older sister and two younger brothers. Her father graduated from Nagoya University School of Medicine with an M.D., and then, for his Ph.D., studied biochemistry at the Biochemistry Department. He moved to clinical medicine and practiced as a medical doctor specializing in obstetrics and gynecology. During the war, he lost his hospital in Nagoya, after which he started working as a director of a hospital in Gamagori, a small city near the beach, not far from Nagoya. As a junior high school student, Tsuneko often visited there to enjoy swimming and looking through a microscope. She graduated from Aichi Prefectural Asahigaoka Senior High School, where the coeducation of boys and girls had been introduced after the war. Her father hoped she would become a doctor, but she chose to study biology at Nagoya University instead. There, starting in 1952, she belonged to the first generation of Japanese women who could study at university together with men. In 1956, she earned her bachelor’s degree, and, in that same year, married Reiji Okazaki, who was three years older and from Hiroshima, and worked in the same group in the Developmental Biology Laboratory.

## A hard-working couple

Tsuneko started working with Reiji (Figure 1), and their passion was DNA. This was the era in which many exciting fundamental discoveries about DNA were made, such as the conclusion in 1953 by Watson and Crick that DNA is structured as a double helix.^3^ In those years, Japan was poor, and even the roof of the laboratory where Tsuneko and Reiji were working leaked. From their private money—testimony to their determination—they bought an expensive piece of equipment (a fraction collector driven by a balancing mechanism). Their persistence paid off, and their first research results included the discovery of thymidine-diphosphate rhamnose, a sugar-linked nucleotide (e.g., reference).^4^ While continuing to work with Reiji, Tsuneko got her Master’s and Ph.D. degrees from Nagoya University in 1958 and 1963, respectively. The quality of their work won them a Fulbright travel grant to the USA, where their visit in 1960–1963 (see below) gave them a first-class preparation for their later groundbreaking discoveries.

## Visiting the USA

As young teenagers, Tsuneko and Reiji grew up during the war. Reiji, at age 15, was in Hiroshima when the first atomic bomb was dropped. Therefore, it may not have been easy for them to visit the USA, the country of their former enemies. Nevertheless, they made life-long friends there, which is evidence of their big hearts. They first stayed 15 months, in 1960–1961, at Washington University, St. Louis, in the lab of Professor Jack L. Strominger (born 1925). Here, they investigated novel nucleotide-linked sugar compounds^5–8^ and learned molecular biology techniques such as dealing with microliter volumes—which was new in those days. Then, mostly traveling on Route 66, they moved from St. Louis to Palo Alto, California. There, at the Stanford University School of Medicine from 1961–1963, they worked for 15 months under the supervision of Professor Arthur Kornberg (1918–2007). Kornberg had discovered DNA polymerase (DNA polymerase I in *E. coli* bacterium) and its function in 1956,^9^ winning the Nobel Prize in Physiology or Medicine in 1959.^10^ The entire Kornberg family was smart and, in 2006, his son Roger would earn a Nobel Prize in Chemistry.^11^ Kornberg’s wife Sylvy was also an excellent scientist and worked closely with her husband, contributing significantly to the discovery of DNA polymerase. This must have been a very stimulating environment for Tsuneko and Reiji to continue their path in science. As they already did in Japan and in the Strominger lab, they continued working on nucleotides in bacteria, but now also included DNA polymerase in their studies.^12,13^ T.N. (Toshi)—an author of this article, who graduated from Nagoya University School of Medicine with an M.D. and, after acquiring his Ph.D. in neurochemistry, then moved to the Department of Biochemistry to continue basic neuroscience research—met with Tsuneko and Reiji for the first time in the Lab of Professor Kornberg to transfer photos of the Okazaki family by the request of Tsuneko’s father, who was his senior at the Department of Biochemistry at Nagoya University School of Medicine. At that time, Toshi and his wife Ikuko visited the USA to work as a Public Health Service Postdoctoral Fellow and a Guest Scientist, respectively, at the NIH. Toshi and Ikuko were highly impressed upon seeing the brilliant and hard work of Tsuneko and Reiji.

## The Question

**Figure 4:**
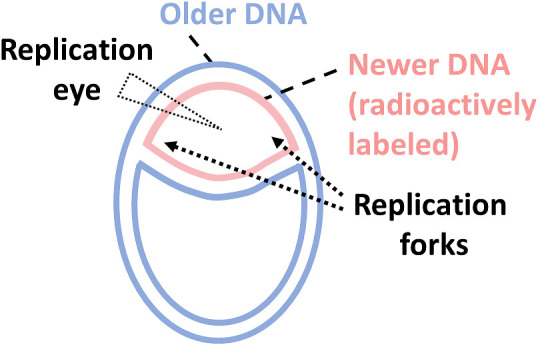
In 1963, John Cairns^14^ found that DNA synthesis involves replication forks, in which the two new strands appear to be simultaneously synthesized. A short period of radioactive labeling in bacteria of newly synthesized chromosomal DNA resulted in a radioactive “eye” structure (pink) whereas longer labeling resulted in a “theta” structure (pink plus blue).

In March 1963, the same month in which the couple returned to Japan where Reiji accepted a position as an Associate Professor at Nagoya University, the question that would become the center of their scientific lives was formulated. In that month, Professor H. John F. Cairns (1922–2018) showed that DNA replication involves simultaneous replication of both DNA strands at a moving locus called the “replication fork,” producing “replication eyes” visible by autoradiography (Figure 4).^14^

**Figure 5:**
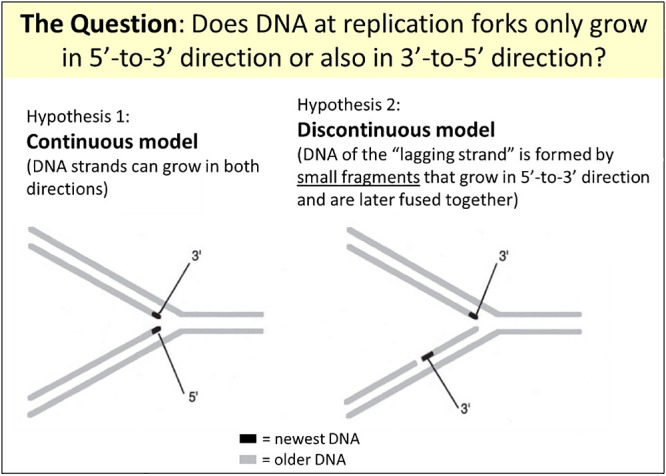
The question was whether DNA replication forks (see Figure 4) are (Hypothesis 1) caused by a continuous extension of both the leading and lagging strands, the latter requiring an unknown 3'-to-5' type of synthesis, or (Hypothesis 2) involve the discontinuous synthesis in a 5'-to-3' direction of short lagging strand fragments that are later ligated together. The figure uses parts of Figure 1 in reference.^1^

The two DNA strands in a helix have opposing directions, which are named after the numbering of the carbon residues in the sugars of the sugar-chain backbone as 5'-to-3' and 3'-to-5' (Figure 3). When new strands are synthesized in each arm of the replication fork, as shown by Cairns in 1963,^14^ essentially one new strand (the leading strand) must be synthesized in the 5'-to-3' direction and the other (the lagging strand) in the 3'-to-5' direction. However, the DNA polymerase activity known at the time could only synthesize DNA in the 5'-to-3' direction. Therefore, there were two possibilities (Figure 5): **(1) *****The continuous model:*** there was an undiscovered DNA polymerase activity that could synthesize DNA chains in the 3'-to-5' direction; or **(2) *****The discontinuous model:*** on the template strand for the lagging strand, first, single-stranded stretches were created that were then copied into lagging strand fragments by the already known 5'-to-3' DNA polymerase activity. Hypothesis 1 or 2 was the question, and the Okazakis concentrated most of their research on 2, after their radioactive labeling experiments only had found evidence for 5'-to-3' and not for 3'-to-5' growth of DNA chains.

## Internationally-winning speed in Japan

THE RACE WAS ON! Here was a question (Hypothesis 1 or Hypothesis 2) that the worldwide DNA research community was interested in, and Tsuneko and Reiji returned to—in those days—underfunded Japan to be the first to answer it. Most Japanese scientists who became internationally famous did so while working abroad or on topics with relatively little competition, but the Okazakis outcompeted the rest of the world on a popular topic largely working in Japan except for another six-month stay in the USA in 1967.

They probably could work so fast because they fully understood each other so that “two people become like three” (we hope that readers are familiar with this magnificent feeling). Besides, they appear to have possessed: (1) A high technical ability for doing biochemical experiments; (2) A deep understanding of the overall question and an experimental focus on the correct model (the discontinuous model of DNA replication); and (3) An exceptionally clever knack for developing experiments to answer their questions. These factors were amplified by their choice of proper organisms for this type of analysis, being bacteria and their viruses (phages), and by having made friends worldwide who provided them with novel mutant bacterial and phage strains that helped answer their questions. This resulted in their international glory in 1968, when in February they published their data supporting the discontinuous DNA replication model in the journal Proceedings of the National Academy of Sciences USA^15^ while in June, Reiji presented the data at the Cold Spring Harbor Symposium on the Replication of DNA in Micro-organisms.^2^

For a more technical and detailed explanation of the discovery of the Okazaki fragments, we refer to the wonderfully written personnel recollection by Tsuneko Okazaki.^1^

## Tragedy strikes and the discovery of RNA priming

However, a major question remained in their model. It was known that DNA polymerase could not start a new DNA strand by itself but could only make an existing strand longer. So, how could Okazaki fragment synthesis be initiated? An initial idea shared by many—and later found to be wrong—was that, at the replication fork, the DNA polymerase would “jump” from synthesizing the leading strand to synthesizing the lagging strand, after which somehow the connection between them would be cut.

Then, in other systems, it became clear that RNA polymerase could synthesize de novo short RNA strands to complement single-stranded DNA and that these nascent RNA strands could serve as “primers” for further elongation by DNA polymerase to produce a DNA strand complementary to the DNA template strand.^16,17^ Reiji and Tsuneko felt that these observations might also explain Okazaki fragment synthesis (e.g., reference),^18^ but they encountered technical difficulties in proving this hypothesis.

Then, tragedy struck. Most probably because of his exposure to the atomic bomb in Hiroshima as a teenager, Reiji developed leukemia. For a long time, he kept working and, despite knowing of his disease, he presented their data in the spring of 1975 in Canada and the USA. He died on August 1, 1975, only 44 years old. Pictures of his life show how brave, kind, and full of energy he was, and this is also how T.N. remembers him.

After Reiji’s death, Tsuneko continued their research. In carrying on, she was supported by a letter from her friend, Nobel Prize laureate Arthur Kornberg, who encouraged her to not give up her important research. She was also supported by her excellent group members, among whom included Professor Emeritus Yoshikazu Kurosawa (Fujita Health University) and the forementioned Professor Fuyuhiko Tamanoi, who were students at the time. In the last period of Reiji’s life, along with later studies by Tsuneko with her students, they gradually overcame the technical difficulties by conducting a set of clever experiments to prove that the synthesis of Okazaki fragments was seeded from RNA primers indeed (Figure 3).^19–22^

After this period, including the 1980s, Tsuneko continued working on the elucidation of DNA replication (e.g., references).^23–30^ In the process, she provided additional experimental evidence for discontinuous DNA strand synthesis, thereby also rebutting some questions raised in the field.

## Women in Science

In 1983, Tsuneko became the first female full Professor at Nagoya University. While a scientist, she was also responsible for taking care of her two children, born in 1963 and 1973—although she did have private help with that, for which she has expressed her sincere gratitude. These challenges inspired her to participate in a citizen’s campaign demanding an improvement of the nursery system.^31^ T.N., co-author of this article, and Dr. Masashi Ikeno, a former group member (now an Associate Professor at Aichi Medical University, Nagakute, Japan) who was interviewed for this article, both recall how Professor Okazaki has always been very supportive of other women in science, including female students. They also remember her saying that at the beginning of her career it was “hard to be a woman.”

## Artificial Chromosomes and Fujita Health University

Later work by Professor Okazaki remained focused on genetic topics, including, for example, the organization of centromers,^32^ regulation of HLA-G expression,^33^ and a mouse model for Down Syndrome.^34^ Perhaps the most spectacular research in the second part of her career was the development of a mammalian artificial chromosome (MAC) system (e.g., references).^35–38^ Professor Okazaki started research on artificial chromosomes in 1989, and it has succeeded in its main target, namely the creation of a convenient system for studying chromatin structure and chromosome functions such as DNA replication and chromosome segregation. For the construction of a MAC, she and her group introduced telomere repeats and selectable markers into a 100 kb yeast artificial chromosome (YAC) containing human centromeric DNA (Ikeno et al., 1998, *Nature Biotechnology*).^35^ When these modified YACs are transfected to mammalian cells, MACs are generated and stably inherited in offspring cells even without selection pressure.^35^ As for gene manipulation, an advantage of a MAC system is that multiple genes (e.g., for a biological pathway) can be introduced simultaneously into a cell in fixed ratios and—using the same MACs—into multiple different cell types. There is still research to date dedicated to MACs (e.g., references)^39,40^ and it is not unfeasible that MAC research will become more prominent in the future, including in the gene manipulation field. However, considering some of its initially very promising aspects for gene manipulation, the MAC system has kind of become “the road not taken” because of the emergence of the extremely successful CRISPR/Cas9 method.

Dr. Masashi Ikeno, who would eventually become her right hand in the MAC studies and who helped to write this article, met Professor Okazaki for the first time in 1989 and was a student in her group at Nagoya University. That group then included the staff members Dr. Tohru Ogawa, Dr. Hisao Masukata, Dr. Hiroshi Masumoto (who at the time managed the studies on artificial chromosomes), and Dr. Kinya Yoda.

**Figure 6:**
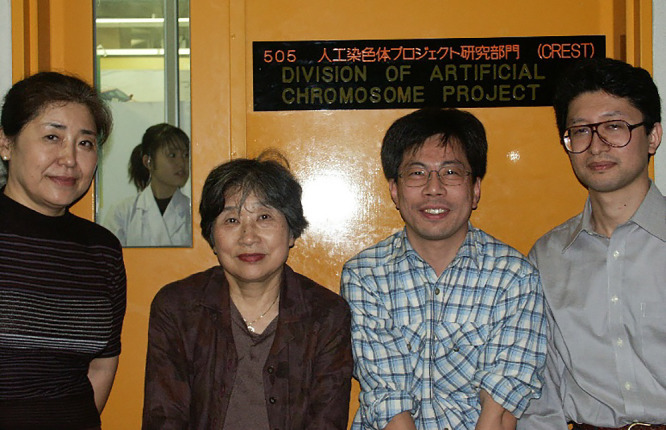
Professor Okazaki and her group, in 2002, in her ICMS lab on the 5^th^ floor in building No. 4 of FHU. From the left: Dr. Kazuyo Yamada, Junko Hotta (behind the window), Professor Okazaki, Dr. Toshihide Itou, and Dr. Masashi Ikeno. The photograph was kindly provided by Dr. Ikeno.

In 1997, Professor Okazaki retired from Nagoya University and continued her research at Fujita Health University, located in Toyoake, a small city adjacent to Nagoya. T.N. and Professor Yoshikazu Kurosawa, one of her former students, helped her with this move. In the same year, she also received a large government “CREST” grant for continuing her artificial chromosome research in 1997–2002. At FHU, she worked at the Institute for Comprehensive Medical Science (ICMS) of which, in 1997, T.N. was the Director. Professor Kurosawa, then a group leader at ICMS, dedicated great efforts to preparing the 5^th^ floor of the ICMS building (FHU building No. 4) for Professor Okazaki (Figure 6) and in 2008 would help her as well to set up a venture company “Chromo Research Inc.” in which Dr. Ikeno also participated. In 2007–2010, Professor Kurosawa became the Director of ICMS and in 2011–2014 the President of FHU.

When Professor Okazaki moved to FHU in 1997, Dr. Ikeno came with her and became an Assistant Professor in her group. In addition, through the CREST project, three postdoctoral researchers were hired: Dr. Toshihide Itou, Dr. Tokuichiro Abe, and Dr. Keiji Hashimoto.

## Stockholm

From 2002–2007, Professor Okazaki served as the Director of the Japan Society for the Promotion of Science, Stockholm Office, allowing her to be closer to her daughter who had married a Swedish man. In Stockholm, at the 2006 Nobel Prize celebration ceremony at the Karolinska Institute, she met again with the Kornberg family when Dr. Roger Kornberg, the eldest son of Arthur, was awarded the Nobel Prize in Chemistry. In her written summary of the event, she recalls how Roger as a high school student would visit their lab at Stanford University, which was what he had asked from his father as a Christmas present.^41^

**Figure 7:**
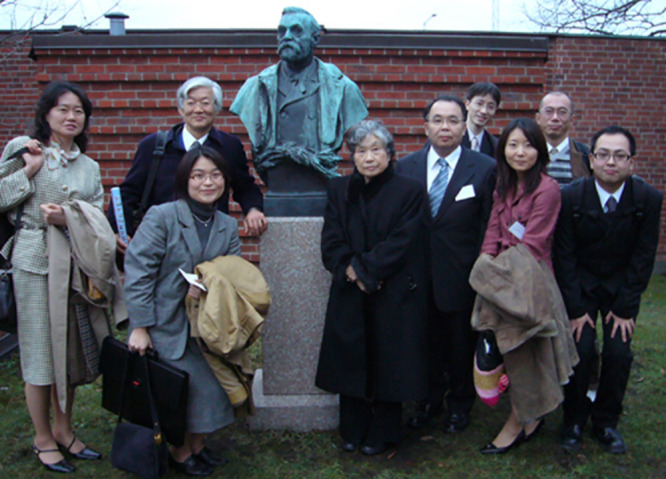
Professor Okazaki and members of FHU besides a bust of Alfred Nobel at the Karolinska Institute, Stockholm, 2007, where they joined a symposium. From the left: Dr. Nobuko Oshima, Professor Yoshikazu Kurosawa (back), Dr. Noriko Satoh (front), Professor Tsuneko Okazaki, Professor Tatsuro Mutoh, Dr. Yoichi Nakajima, Dr. Kanako Koishi, Professor Hiroki Kurahashi, and Dr. Masashi Nakatani. The photograph was kindly provided by Professor Kurahashi.

During this period, Professor Okazaki would come to Japan once or twice a year for a month or so, where she was appointed as a Visiting Professor at FHU. The photograph in Figure 7 shows her with other members of FHU, who, in 2007, visited Sweden for a symposium at the Karolinska Institute.

## Personal memories of people who worked with Professor Okazaki

**Figure 8:**
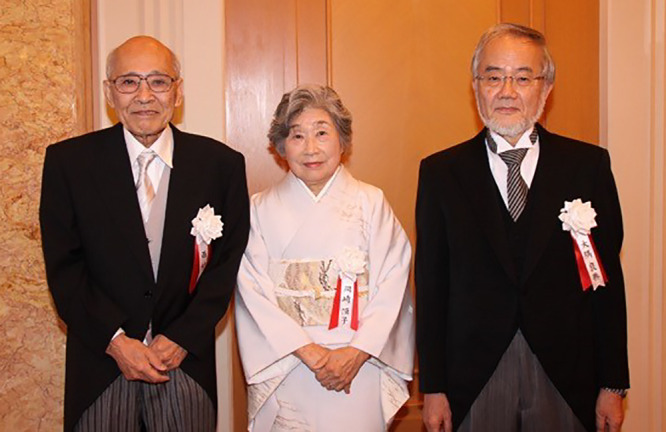
Professor Okazaki at the Person of Cultural Merit Award Ceremony in 2015, together with two of the other prize winners the late Professor Susumu Nishimura (1931–2022) (left) and the Nobel Prize laureate Professor Yoshinori Ohsumi. This photograph was provided courtesy of the President of the RNA Society of Japan, Prof. Shinichi Nakagawa (see https://www.rnaj.org/notice/official-info/item/245-bunkakorosha).

A recurring description of Distinguished Professor Emeritus Tsuneko Okazaki when talking with various people who knew her is the word “elegant,” which can readily be agreed on when seeing photographs (e.g., Figure 8). T.N. and Dr. Ikeno both took Professor Okazaki into their hearts, and remember how much she valued real science, scientific precision, and integrity, and did not care much for science politics. According to Dr. Ikeno, to some people—at first—Professor Okazaki may have given the impression of a reserved person, but when you got to know her and she trusted you, she (when at Fujita after her retirement from Nagoya University) was like a very warm “chatty grandmother” with a slightly mischievous wit, and during their daily breaks the two of them used to talk about all things of life. He also says that she took good care of her group members, and, for example, made sure that all the students in her group would graduate. She also gave her group members a lot of freedom to follow their own scientific creativity. Dr. Ikeno recalls that Professor Okazaki’s hobbies were collecting Japanese antiques and working in her vegetable garden, and that she preferred black tea over coffee.

## Prizes

It is rumored that if Professor Reiji Okazaki had lived, the finding of discontinuous DNA strand synthesis may have been awarded the Nobel Prize. During his life, in Japan, in 1970 and 1971 respectively, he received the prestigious Naito Memorial Prize for the Promotion of Science（内藤記念科学振興賞）and the Asahi Prize（朝日賞）for outstanding accomplishments in the fields of academics and arts. However, the recognition from major prize committees that Professor Tsuneko Okazaki has been a great scientist in her own right and contributed critically and substantially to unraveling the mechanism of DNA replication, came much later. In 1986, she received the Chunichi Culture Award （中日文化賞）; in 2000, the L’Oréal-UNESCO Award for Women in Science; in 2000, The Medal with Purple Ribbon（紫綬褒章）; in 2008, The Order of the Sacred Treasure, Gold Rays with Neck Ribbon（瑞宝中綬章）; in 2015, she was Elected as a Person of Cultural Merit（文化功労者）(Figure 8); and in 2021, she received the Order of Culture（文化勲章）.

However, undoubtedly, the biggest prize of all is that students across the world learn the name Okazaki, which carries the story of how Tsuneko and Reiji together solved one of the important fundamental puzzles in science.

## Figures and Tables

**Table1:** Curriculum Vitae of Distinguished Professor Emeritus Tsuneko Okazaki

1933	Born in Moriyama Ward, Nagoya, as Tsuneko Hara
1952–1963	Department of Biology, Faculty of Science, Nagoya University
	1956, B.S.; 1958, M.S.; 1963, Ph.D.
1956	Marriage to Reiji Okazaki
1960–1961	Fulbright Fellow, Washington University, St. Louis, USA, group of Professor J.L. Strominger
1961–1963	Fulbright Fellow, Stanford University, Palo Alto, USA, group of Professor A. Kornberg
1965–1976	Assistant Professor, Graduate School of Science, Nagoya University
1967–1968	Visiting Assistant Professor, Kansas State University, Manhattan, USA, group of Professor K.G. Lark
1976–1983	Associate Professor, Graduate School of Science, Nagoya University
1983–1997	Professor, Graduate School of Science, Nagoya University
1997–present	Professor Emeritus, Nagoya University
1997–2002	Professor, Institute for Comprehensive Medical Science, Fujita Health University
2002–2012	Visiting Professor, Institute for Comprehensive Medical Science, Fujita Health University
2004–2007	Director of the Japan Society for Promotion of Science, Stockholm Office
2008–2014	President and CEO of Chromo Research Inc.
2016–present	Professor and member of the Institute for Advanced Research (IAR), Nagoya University
2016–present	Distinguished Professor Emeritus, Fujita Health University
